# Development of a Comprehensive Household Food Security Tool for Families with Young Children and/or Pregnant Women in High Income Countries

**DOI:** 10.3390/ijerph191710543

**Published:** 2022-08-24

**Authors:** Amber Bastian, Courtney Parks, Fiona H. McKay, Paige van der Pligt, Amy Yaroch, Sarah A. McNaughton, Rebecca Lindberg

**Affiliations:** 1 Institute for Physical Activity and Nutrition (IPAN), School of Exercise and Nutrition Sciences, Deakin University, Locked Bag 2000, Geelong, VIC 3220, Australia; 2 Gretchen Swanston Centre for Nutrition, 8401 W Dodge Rd., Omaha, NE 68114, USA; 3 Institute for Health Transformation, School of Health and Social Development, Deakin University, Locked Bag 2000, Geelong, VIC 3220, Australia

**Keywords:** food insecurity, survey, pregnancy, young children

## Abstract

Despite increasing rates of food insecurity in high income countries, food insecurity and its related factors are inconsistently and inadequately assessed, especially among households with young children (0–6 years) and pregnant women. To fill this gap, researchers from the U.S. and Australia collaborated to develop a comprehensive household food security tool that includes the known determinants and outcomes of food insecurity among parents of young children and pregnant women. A five-stage mixed methods approach, including a scoping literature review, key informant interviews, establishing key measurement constructs, identifying items and scales to include, and conducting cognitive interviews, was taken to iteratively develop this new comprehensive tool. The resulting 78-item tool includes the four dimensions of food security (access, availability, utilization, and stability) along with known risk factors (economic, health, and social) and outcomes (mental and physical health and diet quality). The aim of this novel tool is to comprehensively characterize and assess the severity of determinants and outcomes of food insecurity experienced by households with young children and pregnant women.

## 1. Introduction

Food insecurity impacts millions of people globally; nearly one in three people did not have access to adequate food in 2020; an increase of 320 million people in just one year [1]. While there has been a significant focus on hunger and food insecurity in low- and middle-income countries, there is an increasing recognition that ‘hidden hunger’ and food insecurity also exist in high income countries (HICs). In Australia, an estimated 3.4 million people (13.5% of the population) experience food insecurity [2]. In the United States (U.S.), an estimated 13.8 million households (10.5% of the population) are impacted [3]. In both countries, there is an inconsistent and inadequate assessment of food insecurity and related factors that families experience which hampers effective programmatic and policy responses.

According to the Food and Agricultural Organization of the United Nations (FAO), achieving food security rests on four dimensions. The first is the provision of enough food to support a healthy lifestyle (availability), the second is food being economically and physically accessible to all people (accessibility), the third is whether people are able to utilize the food they acquire (utilization), and the fourth is how stable the availability, accessibility, and utilization of food is (stability).

There is considerable interest, debate, and uncertainty surrounding the best way to measure food insecurity in HICs. Ashby et al. examined food insecurity measurement tools utilized in this context, assessing the extent to which these tools capture the four dimensions of food security: access, availability, utilization, and stability [4]. The review found that most tools only measured the access dimension, meaning that existing tools are unable to determine the ability of a household to be food secure beyond their ability to physically access and financially afford food. McKay et al. examined how food insecurity has been measured in the Australian context over the last fifteen years, revealing that most tools utilized only measured the access dimension of food insecurity [5]. The U.S. Department of Agriculture (USDA) Household Food Security Survey Module is widely used and validated in the U.S. context, however, this tool is limited as it also only assesses the financial access dimension of food security [4].

The determinants of food insecurity are multifactorial and can occur across multiple levels of the social-ecological model: intrapersonal, interpersonal, organizational, community, and public policy [6]. Determinants of food insecurity include poverty, social and economic disadvantage, individual characteristics (e.g., gender, ethnicity), and the impact of the political and social environment [7]. However, given the limited focus on the access dimension of food insecurity in the most common measurement tools, many estimates of food security do not capture these additional determinants. In addition, most research to date has focused on the general population and, as a result, an understudied population with regard to food insecurity are the parents of young children (ages 0–6) and pregnant women, who are known to experience negative consequences of food insecurity [8,9].

With a global focus on improving maternal and child nutrition through the 2030 Sustainable Development Goals, it is important to understand the experience of food insecurity among pregnant women and families with young children, as food insecurity during these life stages can have ongoing negative health consequences. Women are at risk of becoming food insecure due to entrenched societal power inequality and a range of socio-economic conditions such as domestic violence, poor employment, and education [10,11]. Households with children are at increased risk of food insecurity, as such, mothers and, in particular, single mothers experience a higher rate of food insecurity compared to women without children [11]. Living in a food insecure household during pregnancy may increase the risk of excess gestational weight gain, disordered eating, chronic disease, and various pregnancy complications [12]. The impact of food insecurity on young children is particularly concerning given they are at a key stage of growth and development which can influence health during adolescence and even adulthood [13]. It is important to understand food insecurity among families with young children and pregnant women in HICs as there is limited evidence that examines factors associated with food insecurity among these populations. Identifying such factors is essential to understanding how and when strategies that specifically target food insecurity in this population group may be implemented.

To fill this gap, researchers from the U.S. and Australia collaborated to develop a comprehensive household food security tool that includes the known determinants of food insecurity among parents of young children and pregnant women and to extend existing tools that measure the access dimension of food security. The aim of this study was to develop a new comprehensive household food security tool to characterize and assess the severity of determinants and outcomes of food insecurity experienced by households with young children and pregnant women.

## 2. Materials and Methods

A five-stage mixed methods approach was undertaken to iteratively develop a comprehensive household food security tool. Deakin University provided human research ethics approval (2020-038) and included the University of Nebraska (0642-20-EX). The study was designed and has been reported in accordance with the COREQ checklist for qualitative studies [14]. The first two stages of this study are summarized below and have been published with full details available [15,16] with the next three stages reported in full detail for the first time below.

**Stage 1**

In Stage 1, a scoping literature review was conducted to identify the factors associated with food insecurity among pregnant women and households with young children aged 0–6 years living in HICs. Scoping reviews can be used to identify knowledge gaps, scope a body of literature, and identify key characteristics or factors related to a concept [17]. The scoping review was conducted to investigate factors that influence food insecurity among pregnant women and households with young children (aged 0–6 years) in HICs.

A comprehensive systematic search informed by Peters et al. [18] was conducted in four databases: Medline complete, Embase, Global Health, and CINAHL. These databases were chosen to provide coverage of public health nutrition and nursing and allied health literature in HICs. Search terms were relevant to food insecurity, determinants, pregnancy, and family. The search strategy involved combining the search terms and all terms were searched in the title and/or abstract.

Inclusion criteria were original research articles published in peer reviewed journals in the English language conducted with families or households incorporating pregnant women and/or caregivers of young children aged 0–6 years in high income countries (defined by the Human Development Index). Outcome measures included food security (measured in any way) and/or other aspects of food security as defined by the Food and Agriculture Organization (FAO) such as affordability, access, utilization, or stability.

Relevant information was extracted using predetermined categories including country of study, setting/population, study design, food insecurity measurement tool(s) utilized, and outcomes. Consistent with the approach taken in scoping reviews, articles were not assessed for quality [17]. The range of factors was identified and grouped into 13 overarching constructs. Detailed methods and findings of this scoping review have been published elsewhere [15].

**Stage 2**

In Stage 2, qualitative interviews were conducted with 41 pregnant women or households with young children under 6 years of age who were experiencing or who were at risk of food insecurity in Omaha, U.S. (*n* = 19) and Melbourne, Australia (*n* = 22). A detailed description of the methods and results for these qualitative interviews have been reported [16].

A multi-faceted approach was used to recruit participants. Nutrition and food organizations in Melbourne and Omaha were invited as recruitment sites. One maternity hospital in each city was also approached along with federally subsidized preschools in Omaha and online groups for Melbourne mothers. For those willing to assist with recruitment, fliers were provided to distribute via social media platforms and/or in hard copy. Potential participants contacted the researchers and were provided further information and telephone/online interviews via Zoom [19] were arranged when convenient.

A semi-structured interview guide was based on the findings of the scoping review (stage 1) and designed to gain perspectives on the constructs that influence household food security, key coping strategies, and social, economic, and health conditions that buffer from or exacerbate the experiences of food insecurity. The questions invited participants to provide information about their households (including children, partners, and other household members) and their experiences of feeding a family on a budget. Additional probes were used for pregnant women to elicit pregnancy-specific outcomes.

NVivo 12-assisted coding [20] was completed to establish themes and subthemes from the data, informed by Braun and Clarke’s six-step process [21]. First, all interviews were transcribed iteratively by professional services in each city and checked for accuracy. Secondly, initial codes were developed in line with the interview guide topics and with input from all authors. Authors met regularly to discuss coding and emerging insights and to identify data saturation.

**Stage 3**

Stage 3 involved mapping the themes and subthemes from the qualitative interviews to the constructs found within the scoping review. One researcher (A.B.) conducted the initial mapping which was reviewed by co-authors (C.P., F.H.M., P.v.d.P., and S.M.). Further analysis of the food insecurity themes and constructs was conducted by comparison with known food security frameworks [15,22] to explore how the themes and constructs aligned. Discussion and a consensus on themes and constructs were reached amongst co-authors.

A conceptual basis for the tool was agreed upon by organizing the mapped food security constructs and themes into three components of the household food insecurity experience relevant to families with young children and pregnant women. These were (i) individual or household risk factors for food insecurity (e.g., economic, health, and social), (ii) the four dimensions of food security (e.g., access, availability, utilization, and stability), and (iii) the health outcomes or consequences of food insecurity.

**Stage 4**

In Stage 4, a comprehensive multi-dimensional measurement tool was drafted based on approaches used previously in public health, allied health, and medicine, including reviewing the literature, identifying and/or writing items, and subsequent field testing [23].

Using the agreed conceptual basis (i.e., risk factors, dimensions, and outcomes), appropriate items to include in the tool were identified from reviewing the literature and current measurement tools. These items were then reviewed against the following criteria for selection: use of validated instruments for the risk factors, dimensions, and outcomes of interest; use of brief or short items to reduce participant burden; and use of items used commonly in Australia or the U.S. and/or tested in this population group and/or would allow comparison with national health surveillance.

An extensive list of possible items to include in the tool was drafted and refined by assessing each item against the selection criteria. If no existing items could be found for a construct or theme, the research team developed item(s) taking into consideration the factors suggested by de-Vet et al. [23] for item development, including target population, the purpose of measurement, the difficulty of the items, the application in practice, and response options.

**Stage 5**

In Stage 5, cognitive interviews using the drafted tool were conducted with pregnant women or households with children aged 0–6 years who were experiencing or at risk of food insecurity in Melbourne, Australia (*n* = 11). Budget constraints limited the piloting to one setting. Cognitive interviewing is a psychologically oriented method for empirically studying the ways in which individuals mentally process and respond to survey items [24]. It can be useful in pretesting items and determining how they should be modified to make them easier to understand and answer [25].

Recruitment for the cognitive interviews included contacting participants who had been involved in the semi-structured qualitative interviews conducted in Melbourne (Stage 2) and who had indicated a willingness to be contacted for this additional research component. In total, 9 out of 22 women indicated they were willing to be contacted and 4 agreed to participate in the cognitive interview. The study also was advertised on social media via new mothers’ Facebook groups, and seven women were recruited via this method. Participants completed an online consent form and were contacted by a researcher (A.B.) via phone or email to arrange a convenient interview time.

Interviews were conducted online via Zoom [19] during a two week period (late May, early June 2021) and were, on average, 39 min in length (range from 23 min to 76 min). Interviews were audio recorded and cross-checked against field notes captured by interviewers upon completion of the interview. Participants received $40 in supermarket vouchers as compensation for their time.

During the cognitive interviews, respondents completed the comprehensive household food security tool for families with young children and/or pregnant women with an interviewer while being asked open-ended and probing questions about their responses. Respondents were asked to read and answer the questions out loud and to talk through their decision-making process for each question. This allowed the researcher to discern the participant’s comprehension of items and answer choices.

Respondents were also invited to give general feedback on questions and specific probes were developed for questions by the research team. For example, at the end of the questions on demographics, respondents were asked to reflect and answer the following questions: How hard or easy were these questions to answer? Were any of these more difficult? If so, why? What were you thinking about as you answered? After the question on their main sources of income, respondents were asked: How easy or difficult was it for you to identify your main source of income? Were any of the items confusing or needing further explanation? This allowed for further exploration of how the questions were constructed and suggestions for how these items could be improved.

The interviews were conducted by researchers (A.B. and R.L.). Feedback from cognitive interviews was collated across each item and discussed with co-authors (C.P., F.H.M., and P.v.d.P.). Suggested changes resulting from this feedback were discussed by the research team and the resulting changes and finalization of the tool were agreed upon by all authors.

## 3. Results

The full details of the scoping literature review and qualitative interviews have been published elsewhere (References [15,16]). In brief, findings from the qualitative interviews identified a range of prominent themes associated with food insecurity. The constructs from the scoping review (Stage 1) were mapped against the themes and subthemes arising from qualitative interviews (Stage 2) to identify 13 food security constructs and themes in pregnant women or households with young children aged 0–6 years old (Table 1).

A conceptual basis for the tool was established by organizing the identified food security constructs and themes into three components of the household food insecurity experience. These were organized into: (i) individual or household risk factors for food insecurity (economic, health, and social), (ii) four dimensions of food security (access, availability, utilization, and stability), and (iii) health outcomes or consequences of food insecurity (Table 2).

Using the three identified components described in Table 2, 60 suitable items were identified from the literature and existing measurement tools: 25 items on individual or household risk factors for food insecurity, 13 items on the four dimensions of food security, and 22 items on health outcomes or consequences of food insecurity.

Cognitive interviews with a sample of the population (*n* = 11) led to amendments, resulting in 56 improvements to the measurement tool. These are documented in Appendix A, Table A1. Changes included improving the overall readability (2), changes to question responses offered to make them more relevant (10), changes to question content to make them clearer (22), and adding new items (20) or deleting items (2). Examples of these types of changes are presented in Table 3.

The resulting product is a comprehensive household food security tool for families with young children and/or pregnant women that includes 78 items that span across the three components described above. These can be grouped into 1 screening item, 27 items on individual or household risk factors for food insecurity, 27 items on the four dimensions of food security, and 23 items on health outcomes or consequences of food insecurity.

## 4. Discussion

This study developed a comprehensive household food security tool to examine social demographics and household characteristics, food security status, health, and dietary outcomes for parents and households with young children. The five-stage mixed method research was designed to devise a new tool that would expand the measurement and understanding of household food security beyond financial access. Food security is complex, as illustrated by the many frameworks that attempt to explain its multifactorial determinants and the number of evolving definitions that aim to articulate its various dimensions [6,7]. With our evolving understanding of food security, there is a need to develop and refine measurement tools that capture the diverse aspects of food security. Previous tools such as the USDA household food security survey module, Cornell Child Food Security Measure, Hager two-item screen, and Girard four-point tool have largely focused on the economic or financial aspect of food insecurity [4]. While this is certainly an important pillar of food security, it overlooks the physical accessibility to food, availability of healthy and affordable food, people’s ability to utilize food, and stability of this experience across weeks, months, years, and even generations. This comprehensive household food security tool is unique as it includes items that measure the other dimensions of food security and is specific to households with children under the age of 6 years. Broadly speaking, questions on food literacy and coping skills uncover the ability to utilize food, questions on fruit and vegetable availability and the distance to nearest shops examine the availability dimension, while questions on how various events throughout a year impact food security examine the stability dimension.

The inter-household dynamics between parents/caregivers and their young children are known to be complex in a food insecure context, and hence, a specific tool to investigate these components among an understudied population is warranted. The conceptual basis developed during this research and used to underpin the comprehensive household food security tool highlights the complex nature of food security. Risk factors for food insecurity are commonly attributed to economic variables such as low income and unemployment [26,27,28]. Health risk factors such as maternal depression have also consistently been found to be higher among women experiencing food insecurity [29]. This relationship may be bidirectional as poor mental health has been associated with a transition into food insecurity [30] and food insecurity has been observed to precede depression [31,32]. Further, having a household member with chronic health needs and the associated health care costs is also a risk factor associated with food insecurity [33]. The third type of risk factor contributing to food insecurity is social and includes socio-demographic factors such as ethnicity, education level, marital status, family size, acculturation, and urban life stressors [33,34,35]. The known consequences or outcomes of food insecurity include poorer dietary quality [36,37], associations with diet-related chronic conditions such as diabetes and obesity [38,39], and elevated poor mental health including depression and anxiety [40]. This comprehensive household food security tool could be useful in postnatal, maternal, and child health settings to explore a more comprehensive set of components of food security. Women who report being food secure may in fact present with several economic, health, or social risk factors that, if identified, could enable supports to be implemented in these households to prevent the transition into food insecurity. Furthermore, as the comprehensive household food security tool captures health and dietary outcomes, the inter-relationships between food insecurity and these outcomes can be further explored to better understand the food insecurity experience in families with young children and/or pregnant women.

One strength of this research is that it combines multiple types of evidence into the iterative design of the final instrument. The scoping literature review followed by qualitative and then cognitive interviews with the target population allowed grounding of the tool in both lived experience and evidence. Impacted populations provide their own forms of evidence (knowledge, experience, ideas, and opinions) that aid in expanding the understanding of a given issue [41]. The information gleaned from the qualitative interviews helped to contextualize the constructs found within the scoping literature review to develop the final conceptual framework that underpins the comprehensive household food security tool. Further, the robustness of the tool comes not only from the efforts to generate constructs grounded in people’s daily experiences but also from the efforts to select and generate high-quality items to include in the tool [42].

This tool and study have some imitations. Firstly, the tool is in English. Populations with English as a second language or who do not speak English at all are often at increased risk of food insecurity. Race, ethnicity, and acculturation were identified as factors associated with food insecurity within the literature [27,43], as such, there is a need to develop and test the tool in languages other than English so that food insecurity among diverse populations can be better understood and addressed. Secondly, in 2020, the fifth and sixth dimensions were proposed by the High-Level Panel of Experts on Food Security and Nutrition and include whether people have the ability to make choices and control their engagement with the food system (agency) and whether the food system is environmentally, economically, and socially sustainable (sustainability) (HLPE 2020). As this research commenced prior to the FAO high-level panel of experts recommending the agency and sustainability dimensions, the tool overlooks these two dimensions [44]. Thirdly, while the tool is comprehensive, its length might mean its use is limited to research or to settings where there is sufficient time available to screen.

Future research could explore agency and sustainability and incorporate items around them into the tool to expand the understanding of how these aspects impact food security specifically in this population group. This tool is at its prototype stage and several steps need to be devised before implementing the tool at a population level. These include the development of a scoring system to rate food insecurity status based on social, cultural, and economic risk factors and the severity of food insecurity based on answers to the availability, access, utilization, and stability dimensions of food security. The next step for this measurement tool is reliability and validity testing. Once these steps are undertaken, the tool could be useful in screening families at risk of food insecurity in a range of settings. Future research may also explore how researchers and practitioners may utilize specific scales and items from the comprehensive tool depending on their interests and goals. It could be useful in better measuring and understanding food insecurity in this population group so that policy and programs to address this issue can be devised.

## 5. Conclusions

There is a need for further research beyond the economic dimension of food security to truly understand this complex issue and be able to better identify and support those experiencing food insecurity. Further work is required to test the 78-item comprehensive household food security tool in various settings and populations for reliability and validity. Subsequently, the tool could be used to examine the relationship and increase understanding between risk factors, components of the food insecurity experience, and health outcomes. With an increased understanding of the issue, practitioners, policymakers, and governments will be better placed to identify and implement the required solutions.

## Figures and Tables

**Table 1:** Constructs and themes associated with food security in pregnant women or households with young children aged 0–6 years old.

Food Security Construct	Constructs and Outcomes Identified from Scoping Review (Stage 1)	Themes and Outcomes Identified from Qualitative Interviews (Stage 2)
Income and employment	Low income, job loss, and payment schedules; not receiving welfare; low social economic status (i.e., education, occupation, and household income); living below the poverty line; and mothers as homemakers	Employment; government assistance (e.g., accessing programs, the trade-off between earning income/losing assistance, and running out of assistance); competing expenses (e.g., other bills, children’s activities, time of year, and special occasions)
Coping strategies	Stretching food, going without food, and skipping meals; cutting back on the variety of foods consumed; going to bed hungry; cooking whatever is available, buying cheaper food, shopping at value stores, and using coupons; reducing money spent on children’s education and activities; borrowing money; and food and social supports	Utilizing resources (e.g., food pantries, utility/bill aids, and other non-government programs); budgeting skills (e.g., couponing, bargain shopping, and buying cheaper foods); family and friend support (e.g., food, money, or other resources provided; social support); rationing (e.g., making food last all month, limiting intake); nutrition knowledge and skills (e.g., being a good home cook)
Maternal depression/mental health	Maternal depression and poor health status; parenting stress; lack of time; lack of social support; feelings of isolation; and unwanted childbearing	Stress (e.g., financial stress, stress from children, and stress about feeding children); social factors (e.g., social support or lack thereof, self-portrayal, and stigma); declining mental health contributing to poor food choices; and depression
Residence stability and crowding	Housing and household energy insecurity; experience greater number of moves/relocating; receiving housing subsidy; not owning land; and household crowding	Food utilization; food storage, waste, and kitchen facilities
Education	Caregivers/mothers with lower levels of education	Social demographics (e.g., lower education)
Parent acculturation	Immigrant status and length of time in the country; difficulty with shopping and food preparation in a foreign environment	Social demographics (e.g., foreign students)
Ethnicity	Ethnicity, race, and ethnic minority	Social demographics (e.g., visa status and eligibility for government benefits)
Family composition	Caregiver marital status (single/widowed/separated/divorced); larger household size; and larger number of children	Familial dynamics such as children eating first and children’s awareness of food insecurity; the age of children; and generational food insecurity experience
Health care and Health status	Health care usage; lack of health insurance coverage; poor infant/child health status and greater hospitalizations; high prevalence of overweight/obesity among food insecure children; and children’s behavioral problems	Social demographics (e.g., visa status and eligibility for free or subsidized health care, cost of health care including allied health)
Participation in food assistance programs	Participation in welfare programs (e.g., in federal food assistance programs such as WIC and SNAP in the U.S.); reliance on school meals	Non-traditional food sources (e.g., food pantries, community gardens)
Smoking	Living in a house with a smoker, maternal smoking	Financial impact of competing expenses
Food access and availability	Economic constraints and food pricing (including the cost of fruit and vegetables); choosing between food and other necessities (including medicine and bills); and lack of access to healthy food or food stores in general	Food outlet location; transportation; factors influencing store and item selection; and non-traditional food sources (e.g., dollar stores)
Diet quality	Reduced consumption of high-cost and micronutrient-rich foods; increased consumption of low-cost traditional staple foods	Family food preferences and needs (e.g., priority foods, picky eaters, preferences, dietary needs, culturally appropriate, and pregnancy/toddler/formula needs)
Other	Lack of urban infrastructure and exposure to environmental contaminants	Coronavirus impacts on health, employment and finances, childcare, and food sourcing

**Table 2:** The three components of the household food security experience—a conceptual basis for comprehensively measuring household food security in families with young children and pregnant women.

Components of Household Food Insecurity Experience	
1. Individual or household risk factors for food insecurity	Economic: income and employment Health: stress and mental health, chronic health conditions Social: demographics including education, ethnicity, and household composition
2. The four dimensions of food security	Utilization: resilience and coping strategies, kitchen facilities, nutrition skills and literacy, and participation in food assistance programs Access and availability: physical and financial access to foods and stores, food insecurity screener items Stability: annual competing expenses and challenging times of year
3. Health outcomes or consequences of food insecurity	Health: stress and mental health, chronic health conditions Diet quality

**Table 3:** Examples of changes made to the comprehensive household food security tool resulting from cognitive interviews.

Type of Change	Initial Item in Household Food Security Tool	Change Made to Household Food Security Tool
Improving overall readability	In the past month, about how often did you feel tired out for no good reason?	In the past month, about how often did *you feel tired for no obvious reason?*
Changes to question response to make more relevant	… about how long would it take to get from your home to the nearest local grocery store or supermarket? 1–10 min 11–30 min 31–45 min 46 min–1 h Over an hour Don’t know	Changed responses to: *Less than 5 min* *5–15 min* *16–30 min* *31–45 min* *46 min–1 h* *Don’t know* *Not applicable*
Changes to question content to make clearer	Which category listed below represents the total combined income of all members of your family who are 15 years of age or older. Please include money from things such as jobs, net income from business, pensions, social security payments, and any other income received. Was it…	Which category listed below represents the total combined income of all members of *your household who you share finances (include family members* 15 years of age or older). Please include money from things such as jobs, net income from business, pensions, social security payments, child support and any other income received, *before tax is taken out*. Was it…
Adding or deleting items to make more relevant	Are there times of the year or events where buying food for your household is more difficult due to competing expenses? Please tick any of the below that makes it more difficult for you to purchase food for your household.	Added in a separate/additional item: *Please describe the other times or events when buying food is more difficult (free text response)*
	Do you consider yourself to be an acceptable weight, underweight or overweight?	Deleted question as subjective and survey has question items on self-reported weight and height

## Data Availability

The comprehensive household food security tool can be obtained from the corresponding author upon email request.

## Appendix A

**Table A1:** Changes made to the survey as a result of cognitive interview feedback.

Question Item	Responses Options	Feedback from Cognitive Interviews	Changes Made to Survey
If you are not an Australian Citizen, what visa are you on? Leave blank if not applicable.	Free Text	Interview 1: Could just have ‘yes’ as an option: reword ‘are you an Australian citizen’? Yes, No. If not, add in what visa question; if yes, skip this question	Change as suggested to “are you an Australian citizen or permanent resident”? Yes/No options (A) Follow up question if “No” is selected; “What visa are you on?” free text response option (Q)
Which of the following best describes your housing or living situation? Tick one:	Living with children and partner/spouse Living with children Living with partner/spouse Living with parents/extended family Living by myself Living with flatmates/friends Other (please specify)	Interview 1: For option 2, ‘living with children’ add in ‘without spouse’. Could add into question ‘own or foster’ children Interview 4: Hard to locate answer (living with children)	Changed second response option to “living with children without partner/spouse” (R) Changed wording of question item to “Which of the following best describes who you live with? Choose one”. (Q) Removed wording ‘living with’ from responses as question item wording changed (O)
What is your current living arrangement?	Homeowner Renting (privately) Renting (public housing or community housing) Boarding house or caravan park Temporary accommodation (staying with family or friends, shelters, hostel) Living on the street Other	Interview 1: Add ‘living with parents/family’ as an option as this may be a permanent situation and therefore doesn’t come under the temporary accommodation option Interview 5: Have two options for homeowner: with mortgage and without mortgage	Added new response option “Permanently staying with family or friends” (R) Changed homeowner response options to “Homeowner no mortgage” or “Homeowner with mortgage” (R)
What is your main source of income?	Wages or salary Any government pension or allowance Self funded retirement Nil or negative Don’t know Any other regular source—please answer 14a	Interview 5: Add in an additional question ‘are you the main income earner in the home’ Interview 7: Answer for self or partner?	Changed question item wording to include household so the question now reads “What is your main source of household income”? (Q)
Which category listed below represents the total combined income of all members of your family who are 15 years of age or older. Please include money from things such as jobs, net income from business, pensions, social security payments, and any other income received. Was it…	No income $1–$119 per week ($1–$6239 annually) $120–$299 per week ($6240–$15,999 annually) $300–$499 per week ($16,000–$25,999 annually) $500–$699 per week ($26,000–$36,999 annually) $700–$999 per week (37,000–$51,999 annually) $1000–$1499 per week ($52,000-$77,999 annually) $1500 or more per week ($78,000 or more annually)	Interview 1: What about if living with housemates or parents? Are these included? Word ‘family’ may be confusing as may word ‘household’ Interview 2: Does this include family tax benefit? Would interpret answering this question pre-tax/gross but doesn’t specify Interview 4: Had to stop and really think Interview 6: Most payments are fortnightly Interview 7: Pre or post tax? Interview 8: Do I include child support? Interview 11: Like how there was an annual, this helped to work out more so than the weekly total	Changed wording in this question to include the text “members of your household who you share finances with” (Q) Added text “before tax is taken out” to question (Q) Added text “child support” into question (Q)
Do you receive any government benefits? Yes/No If yes, which ones do you receive? Tick all that apply:	JobSeeker Austudy/abstudy Disability support pension Carer payment Parenting payment Aged pension Rent assistance Other	Interview 1: Add parenting payment? Interview 2: Does parenting payment cover part parenting payment? What about family tax benefit, also under parenting payment? Interview 5: Add in ‘family tax benefit as an option Interview 7: What about childcare subsidy? Does this come under family tax benefit? Interview 11: Think childcare should be here	Included “family tax benefit” as a response option (R) Allow free text if the “other” response option is selected (R)
For the following statements please choose the answer that best fits for the past year: My family has enough money to afford the kind of home we would like to have We have enough money to afford the kind of clothing we should have We have enough money to afford the kind of furniture or household equipment we should have We have enough money to afford the kind of car we need We have enough money to afford the kind of food we should have We have enough money to afford the kind of medical care we should have My family has enough money to afford the kind of leisure and fun activities we want to participate in	Strongly disagree Disagree Neutral Agree Strongly agree	Interview 5: Have ‘adequate’ but not ‘ideal’ for most of these questions Most interviewees struggled to answer this scale as they found it hard to distinguish between essential items (i.e., any home) and what they would like (i.e., a home they would like)	Scale removed (D)
In the last month did (you/you or other adults in your household) ever cut the size of your meals or skip meals because there wasn’t enough money for food?	No Yes—please answer 3a. Don’t know	Interview 2: Asks about adults	Added in an additional eight USDA questions specific to children for respondents who indicate they have children (8A) Added four new questions on children’s health (4A) Added question about children’s awareness of the food insecurity experience (A)
What kind of transport do you usually use to purchase food/groceries? Tick one only	I drive my own car I ride with friends or family I borrow a car I take public transport (train, tram, bus, or combination) I take a taxi or app-based ride like Uber I walk or take my bicycle Other, please specify	Interview 1: What about online grocery delivery (Coles online, Woolworths online)? Interview 2: Would tick two options if could Interview 7: Add in online delivery as option	Created a new question about where people shop that precedes this question about transportation to purchase food/groceries. The new question includes online shopping (A)
Using this usual form of transport listed in the previous question, about how long would it take to get from your home to the nearest local grocery store or supermarket?	1–10 min 11–30 min 31–45 min 46 min–1 h Over an hour Don’t know	Interview 11: In cities most people are going to be less than 5 min, so maybe have 1–5 and 6–10 min	Changed response options: “Less than 5, 5–15, 16–30, 31–45, 46–1h” (R)
For the following questions choose the answer which best fits. How often during the *past month* did you or anyone in your household have to …. choose between paying for food and paying for medical care? choose between paying for food and paying for utilities? choose between paying for food and paying for rent or mortgage? choose between paying for food and paying for transportation or gas for a car? choose between paying for food and paying for school loans, tuition, or other education expenses? stretch the amount of food in your home by limiting the amount of food people in your home could eat? avoided inviting guests into your home when you would be expected to serve them food? eaten meals or snacks *after* your children finished to ensure they had enough? visited a food bank, pantry, or other emergency food relief service	Never Rarely Sometimes Often Always	Interview 5: Does this include medication? Interview 11: Should there be a not applicable here and for all? For us, this question is not relevant for the last month Interview 2: Could include cost of childcare in this question Interview 5: Would include question on pets as often chose to feed pets before self when things were tight with money and cost of their food impacts budget. Also include question on medical expenses (GP/medicine) and allied health (dentist/physiotherapist, etc.)	Added in the text “medication” to the first line item so question item now reads “medicine and/or medical care”? (Q) Added in “not applicable” to response options (R) Added in the text on childcare or after school care (Q) Added in the text about nappies and infant food/formula to be more relevant to children (Q) Added in the text on feeding pets (Q) Added in the text on allied health care (dentist, physiotherapist, psychologist, etc.) (Q) Split question item into two separate items (A)
For the following statements/questions choose the answer which best fits: Meals are an important part of the day for me/my household I am able to cook healthy foods for my family on a budget I am able to cook from basic ingredients I plan meals ahead (e.g., for the day/week ahead) I use leftovers to create another meal I buy food in season to save money I purchase healthy food, even if I have limited money? (e.g., fruit and vegetables) I compare prices between products in order to get the best value food	Strongly disagree Somewhat disagree Neutral Somewhat agree Strongly agree	Interview 1: Don’t create another meal with leftovers but eat leftovers; how to answer this question? Interview 1: Don’t always know what is in season but just buy cheapest foods. Interview 11: What about buying in bulk?	Changed question item wording from “I use leftovers to create another meal” to “I use leftovers” (Q) Added in new free text item “Are there any other food budgeting strategies you use (e.g., couponing, buying in bulk)? Please write below:” (A)
Are there times of the year or events where buying food for your household is more difficult due to competing expenses? Please tick any of the below that makes it more difficult for you to purchase food for your household.	Christmas/Ramadan/other religious festivals School holidays Start of school year/term Birthdays Towards the end of the pay cycle Loss of job Moving house Unexpected car issues Other (please specify)	Interview 2: Add in Easter to first response item, also include winter and increased heating expenses as an option Interview 3: Add in medical care/expenses. Interview 4: Would tick all answers here Interview 5: Include ‘unpaid sick leave’ and also ‘COVID/lockdown’ Interview 6: Could also include homelessness/loss of home Interview 9: Also include reduced work hours as event Interview 11: Not school but when daycare is closed, we do spend more money on feeding my daughter because she is at homeilot C:	Changed first response option to “religious festivals” and provided examples (R) Added in new response options; “Increased heating in winter or cooling in summer, Reduction in work hours, Medical care/medical expenses, Recent death/bereavement, End of a relationship, Delays in Centrelink payments, COVID-19 restrictions, Homelessness, loss of home” (R) Added in new free text item to describe other events or times when buying food is more difficult (A)
Do you currently smoke?	No Yes—please answer 2a	Interview 5: Include question on if partner or other household members smoke	Changed question item wording to “Do you or anyone in your household smoke cigarettes or purchase other tobacco products?” (Q)
If yes, do you currently smoke regularly, that is at least once per day?	No Yes		Added question about frequency of smoking “Do you or your household member currently smoke or vape regularly, that is at least once per day? (Q)
Are you currently pregnant?	No Yes—please answer 4a.	Interview 11: This is so influential in all my answers, maybe it should go at the top? Like I want to answer this first because its context for all my other answers. Same as having a toddler, our budget has changed to ensure her diet is varied and optimal	Have moved this question to the very start of the survey as a new screener question (Q) Added new question on if this is a planned pregnancy (A)
Have you taken any dietary supplements (e.g., multivitamins, fish oil) in the last 24 h?	No Yes Don’t know	Interview 1: Add in ‘folate’	Added “folate” under the examples provided (Q)
Please indicate if you have ever experienced any of the following as an adult or child?	Financial or economic abuse Emotional or psychological abuse Spiritual abuse (the denial or use of spiritual or religious beliefs and practices to control and dominate another person) Physical abuse Sexual abuse Other abuse Prefer not to answer	Interview 1: Need to add into wording ‘leave blank if none apply’ Interview 2: Think this is an important question to include Interview 5: Need to have lead into question indicating it may be sensitive and can choose to skip or not answer if prefer. Interview 11: Have a ‘none of the above’ answer	Changed question item wording to include “tick any that apply” (Q) Added in response option “none of the above” (R) Moved from the start of the survey under the ‘about you’/demographics section to near the end of the survey under the ‘health’ section so not as not to be jarring and added in text prior to the question “Some of the next questions may be sensitive and you can skip them if you prefer not to answer”. (Q)
For the following statements choose the answer which best fits: In the past month, about how often did you… feel tired out for no good reason?	None of the time A little of the time Some of the time Most of the time All of the time	Interview 1: What does ‘not good reason’ mean? i.e., is it a health or lifestyle reason? Hard to answer; need a ‘not sure’ option Interview 4: ‘tired out’ reads strange. Does this mean exhausted? Suggest changing Interview 5: ‘no good reason’ have many reasons Interview 7: ‘no good reason’?	Changed question item wording to “feel tired for no obvious reason” (O)
Do you consider yourself to be an acceptable weight, underweight, or overweight?	Acceptable weight Underweight Overweight Currently pregnant	Interview 5: Would answer ‘slightly’ overweight if there was this option Interview 7: Probably acceptable weight but overweight for pre-baby Interview 11: Yes, doctors tell me I am for this stage in pregnancy	Removed the question as respondents are asked to report weight and height so BMI can be calculated from this information (D)
Over the last month, how many glasses of sugar sweetened beverages (e.g., regular soft drinks like Coca-Cola, Pepsi, Solo, lemonade, sweetened tea, and fruit drinks) did you usually drink each day? Do not include mineral or soda water.	None Less than 1 glass per day 1 glass per day 2 glasses per day (equivalent to 1 can) 3 glasses per day 4 glasses per day 5 glasses per day 6 glasses per day 7 glasses per day (equivalent to a 1.25 L bottle) 8 glasses per day 9 glasses per day 10 or more glasses per day	Interview 2: ‘Fruit drinks’ would include juice in answer for this. Interview 4: Under fruit drink would include juice in calculations Pilot	Included the line saying do not include 100% fruit juice here (Q) Added sports and energy drinks (Q)
Over the last month, how often did you eat fresh meat (including beef, veal, chicken, lamb, pork)?	Never Less than once per month 1–3 times per month 1 time per week 2 times per week 3–4 times per week 5–6 times per week 1 time per day 2 times per day 3 or more times per day	Interview 1: Household is vegetarian so need option for this. Perhaps at the start of the survey could have a question about dietary requirements, e.g., gluten free, vegetarian/vegan, etc. Interview 2: Provide option for vegetarian, i.e., ‘never’ by choice or ‘never’ because can’t afford? Interview 4: Include frozen as well in this answer?	Added in a question prior to this; “Do you follow a vegetarian or vegan diet?” If they select no then they are asked to answer this question, if they select partly (pescatarian) then they are asked to skip this question but answer other questions on fish, if they select yes then they can skip the question (A) Have included the word “frozen” in the question item so the question now reads; “Over the last month, how often did you eat fresh or frozen meat (including beef, veal, chicken, lamb, pork)?” (Q)
Over the last month, how often did you eat take away or fast foods (such as burgers, chips, pizza, Indian)? Include foods eaten at the restaurant or at home (e.g., Uber eats, take away)	Never Less than once per month 1–3 times per month 1 time per week 2 times per week 3–4 times per week 5–6 times per week 1 time per day 2 times per day 3 or more times per day	Interview 5: Would take out Indian as haven’t mentioned other ethnic cuisines.	Have taken out “Indian” from question item wording (Q)

(A) added an item, (D) deleted an item, (Q) changed question content, (R) changed response option, and (O) overall readability improved.
